# Bis{2-meth­oxy-6-[(4-methyl­phen­yl)iminiometh­yl]phenolato-κ^2^ *O* ^1^,*O* ^2^}tris­(nitrato-κ^2^ *O*,*O*′)methano­lsamarium(III)

**DOI:** 10.1107/S1600536811000407

**Published:** 2011-01-15

**Authors:** Hang-Ming Guo

**Affiliations:** a Jinhua College of Vocation and Technology, Jinhua, Zhejiang 321017, People’s Republic of China

## Abstract

The asymmetric unit of the title compound, [Sm(NO_3_)_3_(C_15_H_15_NO_2_)_2_(CH_3_OH)], contains two Schiff base 2-meth­oxy-6-[(4-methyl­phen­yl)iminiometh­yl]phenolate (H*L*) ligands, three nitrate ions and one methanol mol­ecule that binds to the nine-coordinate samarium(III) ion *via* its O atoms. The H*L* ligands chelate with a strong Sm—O(deprotonated phenolic) bond and a weak Sm—O(meth­oxy) contact. The latter can be inter­preted as the apices of the bicapped square-anti­prismatic Sm^III^O_9_ polyhedron. The Schiff base ligands are in a zwitterionic state with the phenolic H atom transferred to the imine N atom. O—H⋯O, O—H⋯N and N—H⋯O hydrogen bonds lend stability to the structure. One O atom of one nitrate group is equally disordered over two positions.

## Related literature

For the syntheses of rare earth complexes with Schiff bases derived from *o*-vanillin and adamantane­amine, see: Burrows & Bailar (1966 ▶); Li *et al.* (2008 ▶); Xian *et al.* (2008 ▶); Zhao *et al.* (2005 ▶); Liu *et al.* (2009 ▶, 2010 ▶). For their applications, see: Leadbeater & Marco (2002 ▶); Quici *et al.* (2004 ▶).
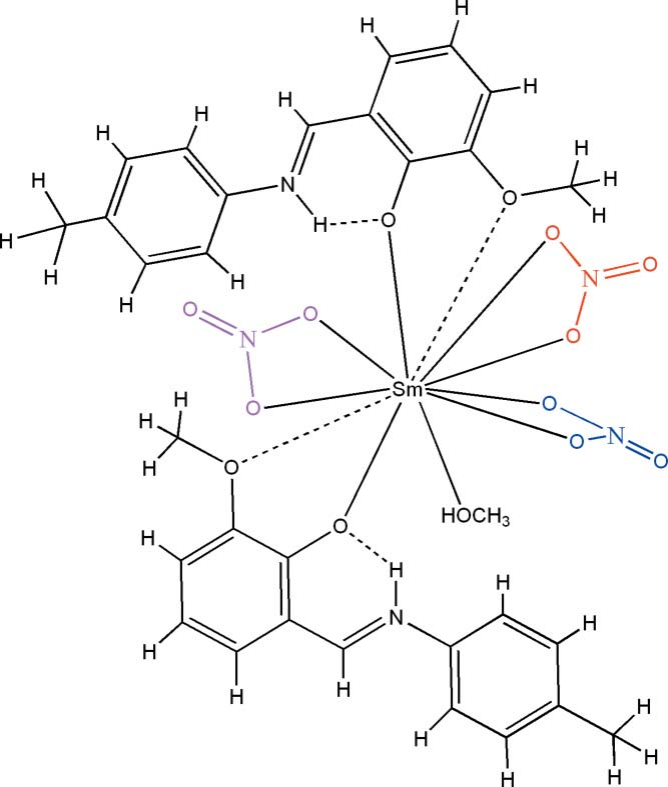


## Experimental

### 

#### Crystal data

[Sm(NO_3_)_3_(C_15_H_15_NO_2_)_2_(CH_4_O)]
*M* *_r_* = 850.99
Triclinic, 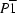

*a* = 7.8547 (10) Å
*b* = 14.6893 (19) Å
*c* = 16.590 (2) Å
α = 73.402 (8)°
β = 85.738 (7)°
γ = 79.230 (7)°
*V* = 1801.6 (4) Å^3^
*Z* = 2
Mo *K*α radiation
μ = 1.70 mm^−1^
*T* = 296 K
0.26 × 0.11 × 0.08 mm

#### Data collection

Bruker APEXII area-detector diffractometer
Absorption correction: multi-scan (*SADABS*; Sheldrick, 1996 ▶) *T*_min_ = 0.797, *T*_max_ = 0.870
25616 measured reflections
6337 independent reflections
5512 reflections with *I* > 2σ(*I*)
*R*_int_ = 0.043

#### Refinement

*R*[*F* ^2^ > 2σ(*F* ^2^)] = 0.043
*wR*(*F* ^2^) = 0.123
*S* = 1.05
6337 reflections
474 parameters
H-atom parameters constrained
Δρ_max_ = 0.91 e Å^−3^
Δρ_min_ = −0.73 e Å^−3^

### 

Data collection: *APEX2* (Bruker, 2006 ▶); cell refinement: *SAINT* (Bruker, 2006 ▶); data reduction: *SAINT*; program(s) used to solve structure: *SHELXS97* (Sheldrick, 2008 ▶); program(s) used to refine structure: *SHELXL97* (Sheldrick, 2008 ▶); molecular graphics: *SHELXTL* (Sheldrick, 2008 ▶); software used to prepare material for publication: *SHELXL97*.

## Supplementary Material

Crystal structure: contains datablocks I, global. DOI: 10.1107/S1600536811000407/bv2165sup1.cif

Structure factors: contains datablocks I. DOI: 10.1107/S1600536811000407/bv2165Isup2.hkl

Additional supplementary materials:  crystallographic information; 3D view; checkCIF report

## Figures and Tables

**Table 1:** Hydrogen-bond geometry (Å, °)

*D*—H⋯*A*	*D*—H	H⋯*A*	*D*⋯*A*	*D*—H⋯*A*
O14—H14*B*⋯O10^i^	0.82	2.04	2.859 (6)	174
O14—H14*B*⋯O8^i^	0.82	2.53	3.121 (6)	130
O14—H14*B*⋯N4^i^	0.82	2.60	3.367 (6)	157
N1—H1*A*⋯O1	0.86	1.96	2.637 (5)	135
N1—H1*A*⋯O6	0.86	2.65	3.449 (7)	154
N2—H2*A*⋯O3	0.86	2.02	2.678 (5)	132
N2—H2*A*⋯O11	0.86	2.52	3.311 (5)	153

Symmetry code: (i) 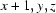.

**Fig. 1.**
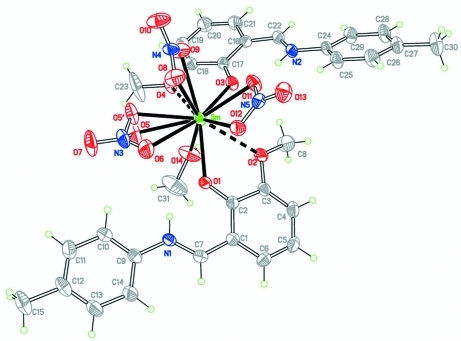
The molecular structure of the title complex, showing the atom-labeling scheme. Weaker bonds to methoxy O donors shown with dashed lines. Displacement ellipsoids are drawn at the 30% probability level.

**Fig. 2.**
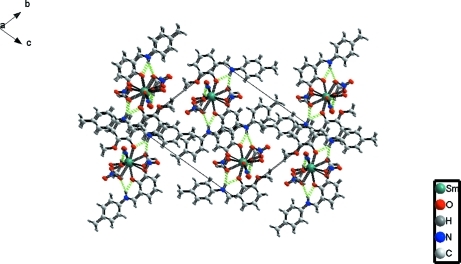
The stacking plot of the title compound, showing H-bond interactions (dashed lines) and π–π stacking interactions.

## supplementary crystallographic information

### Comment

Schiff base complexes utilizing ligands obtained from substituted *o*-vanillin have been attracted considerable attention in past decades due to the intriguing biological activities of *o*-vanillin (Zhao *et al.*, 2005) and its convenience in Schiff base syntheses (Burrows & Bailar, 1966). Interested in this field, we have been engaged in a major effort directed toward the development of syntheses of new analogous Schiff bases derived from *o*-vanillin and their rare metal complexes. In a few of articles we have reported our partial research results (Zhao *et al.*, 2005; Xian *et al.* 2008; Li *et al.* 2008; Liu *et al.*, 2009). Herein, we describe a new Sm^III^ complex.

The structure of the title complex is shown in Fig.1. In this complex, the O5 atom in a nitrate anion is disordered over two sites (assigned in a 50: 50 ratio). The Sm^III^ is nine-coordinated by O atoms, six of which come from three nitrate ions, one from methanol and two from the Schiff base ligands (H*L*). The H*L* ligands coordinate to the Sm^III^ ion using oxygen atoms from deprotonated phenolic hydroxyl groups. Interestingly, the Schiff base ligands are in a zwitterionic state with the phenolic H transferred to the imine N. The bonds between Sm^III^ and O atoms from phenoxy groups are 2.486 (3) and 2.428 (3), which are shorter than those between Sm^III^ and O atoms of methoxyl groups (2.806 (4) Å and 2.957 (4) Å for Sm—O2 and Sm—O4). The nitrate anions coordinate to the Sm^III^ via O atoms with distances ranging from 2.59 (2) to 2.743 (5), which are intermediate between the Sm—O(phenolic) and the Sm—O (methoxy) bond lengths. The Sm—*O*(methoxyl) bond length is only slightly longer than that for Sm—O(phenolic), and these values are similar to those reported for related complexes (Liu *et al.*, 2010).

The hydrogen bonds and π–π weak non-covalent interactions lend stability to the structure. The hydrogen bonds are listed in Table 2 and the stacking plot of this compound is shown in Fig. 2. Different lines are interlocked with benzene rings of Schiff base using π–π stacking. As indicated above, in the H*L* ligands, the proton of the phenolic hydroxyl group has been transferred to the *N*-imine atom, and is involved in an intramolecular hydrogen bond (Table 2).

### Experimental

Reagents and solvents used were of commercially available quality and used without further purification. The Schiff base ligand 2-[(4-methylphenyl)iminomethyl]-6-methoxy-phenol was prepared by condensation of *o*-vanillin and *p*-methylaniline with a high yield and which was purified by recrystallization in ethanol. The compound (1) was obtained by adding Sm(NO_3_)_3_ (1 mmol, dissolved in methanol) to *N*-salicylidene-*p*-toluidine (2 mmol) in methanol solution. The solution was stirred at room temperature for 8 h to obtain a purplish red solution. At last, the deposit was filtered out and the solution was kept for evaporating. Red crystals were formed after several days.

### Refinement

The structure was solved by direct methods and successive Fourier difference synthesis. The H atoms bonded to C and N atoms were positioned geometrically and refined using a riding model [aliphatic C—H =0.96 Å (*U*_iso_(H) = 1.5*U*_eq_(C)), aromatic C—H = 0.93 Å (*U*_iso_(H) = 1.2*U*_eq_(C)) and N—H = 0.86 Å with *U*_iso_(H) = 1.2*U*_eq_(N).

### Crystal data

**Table:**

[Sm(NO_3_)_3_(C_15_H_15_NO_2_)_2_(CH_4_O)]	*Z* = 2
*M**_r_* = 850.99	*F*(000) = 858
Triclinic, *P*1	*D*_x_ = 1.569 Mg m^−^^3^
Hall symbol: -P 1	Mo *K*α radiation, λ = 0.71073 Å
*a* = 7.8547 (10) Å	Cell parameters from 7607 reflections
*b* = 14.6893 (19) Å	θ = 1.7–25.0°
*c* = 16.590 (2) Å	µ = 1.70 mm^−^^1^
α = 73.402 (8)°	*T* = 296 K
β = 85.738 (7)°	Block, red
γ = 79.230 (7)°	0.26 × 0.11 × 0.08 mm
*V* = 1801.6 (4) Å^3^	

### Data collection

**Table:**

Bruker APEXII area-detector diffractometer	6337 independent reflections
Radiation source: fine-focus sealed tube	5512 reflections with *I* > 2σ(*I*)
graphite	*R*_int_ = 0.043
φ and ω scans	θ_max_ = 25.0°, θ_min_ = 1.7°
Absorption correction: multi-scan (*SADABS*; Sheldrick, 1996)	*h* = −9→9
*T*_min_ = 0.797, *T*_max_ = 0.870	*k* = −17→16
25616 measured reflections	*l* = −19→19

### Refinement

**Table:**

Refinement on *F*^2^	Primary atom site location: structure-invariant direct methods
Least-squares matrix: full	Secondary atom site location: difference Fourier map
*R*[*F*^2^ > 2σ(*F*^2^)] = 0.043	Hydrogen site location: inferred from neighbouring sites
*wR*(*F*^2^) = 0.123	H-atom parameters constrained
*S* = 1.05	*w* = 1/[σ^2^(*F*_o_^2^) + (0.0718*P*)^2^ + 2.0645*P*] where *P* = (*F*_o_^2^ + 2*F*_c_^2^)/3
6337 reflections	(Δ/σ)_max_ = 0.001
474 parameters	Δρ_max_ = 0.91 e Å^−^^3^
0 restraints	Δρ_min_ = −0.73 e Å^−^^3^

### Special details

**Table:**

Geometry. All e.s.d.'s (except the e.s.d. in the dihedral angle between two l.s. planes) are estimated using the full covariance matrix. The cell e.s.d.'s are taken into account individually in the estimation of e.s.d.'s in distances, angles and torsion angles; correlations between e.s.d.'s in cell parameters are only used when they are defined by crystal symmetry. An approximate (isotropic) treatment of cell e.s.d.'s is used for estimating e.s.d.'s involving l.s. planes.
Refinement. Refinement of *F*^2^ against ALL reflections. The weighted *R*-factor *wR* and goodness of fit *S* are based on *F*^2^, conventional *R*-factors *R* are based on *F*, with *F* set to zero for negative *F*^2^. The threshold expression of *F*^2^ > σ(*F*^2^) is used only for calculating *R*-factors(gt) *etc*. and is not relevant to the choice of reflections for refinement. *R*-factors based on *F*^2^ are statistically about twice as large as those based on *F*, and *R*- factors based on ALL data will be even larger.

### Fractional atomic coordinates and isotropic or equivalent isotropic displacement parameters (Å^2^)

**Table:**

	*x*	*y*	*z*	*U*_iso_*/*U*_eq_	Occ. (<1)
Sm	−0.01017 (3)	0.327313 (17)	0.806379 (17)	0.04596 (12)	
O1	0.0691 (5)	0.4434 (2)	0.6739 (2)	0.0471 (8)	
O2	0.2571 (5)	0.2701 (3)	0.6999 (3)	0.0583 (10)	
O3	0.1465 (5)	0.1745 (2)	0.8891 (2)	0.0456 (8)	
O4	0.0886 (6)	0.2926 (3)	0.9826 (3)	0.0608 (11)	
O5	−0.109 (3)	0.4813 (14)	0.8558 (16)	0.111 (8)	0.50
O5'	−0.165 (3)	0.4487 (11)	0.8865 (11)	0.073 (4)	0.50
O6	−0.2260 (8)	0.5018 (4)	0.7536 (4)	0.0900 (16)	
O7	−0.3204 (8)	0.5922 (4)	0.8313 (4)	0.109 (2)	
O8	−0.3483 (6)	0.3231 (4)	0.8222 (3)	0.0765 (13)	
O9	−0.2151 (6)	0.2403 (4)	0.9302 (3)	0.0735 (12)	
O10	−0.4885 (5)	0.2504 (4)	0.9299 (3)	0.0859 (16)	
O11	−0.0722 (6)	0.1724 (3)	0.7704 (3)	0.0583 (10)	
O12	−0.1428 (6)	0.3007 (3)	0.6725 (2)	0.0579 (10)	
O13	−0.1868 (6)	0.1644 (3)	0.6599 (3)	0.0726 (12)	
O14	0.2519 (6)	0.3925 (3)	0.8307 (3)	0.0835 (15)	
H14B	0.3311	0.3550	0.8584	0.125*	
N1	−0.0024 (5)	0.6247 (3)	0.5829 (3)	0.0416 (9)	
H1A	−0.0255	0.5804	0.6267	0.050*	
N2	0.2117 (6)	−0.0081 (3)	0.8804 (3)	0.0461 (10)	
H2A	0.1604	0.0499	0.8577	0.055*	
N3	−0.2281 (8)	0.5202 (4)	0.8184 (4)	0.0698 (15)	
N4	−0.3519 (6)	0.2712 (3)	0.8951 (3)	0.0492 (10)	
N5	−0.1351 (6)	0.2118 (3)	0.7001 (3)	0.0478 (10)	
C1	0.1944 (6)	0.5004 (4)	0.5381 (3)	0.0406 (11)	
C2	0.1746 (6)	0.4282 (3)	0.6144 (3)	0.0412 (11)	
C3	0.2795 (7)	0.3353 (4)	0.6224 (4)	0.0469 (12)	
C4	0.3871 (7)	0.3178 (4)	0.5585 (4)	0.0552 (14)	
H4A	0.4532	0.2568	0.5650	0.066*	
C5	0.4003 (8)	0.3892 (5)	0.4837 (4)	0.0570 (14)	
H5A	0.4738	0.3753	0.4406	0.068*	
C6	0.3066 (7)	0.4788 (4)	0.4733 (3)	0.0507 (13)	
H6A	0.3164	0.5264	0.4232	0.061*	
C7	0.1065 (7)	0.5962 (4)	0.5278 (3)	0.0437 (11)	
H7A	0.1276	0.6422	0.4782	0.052*	
C8	0.3849 (10)	0.1852 (5)	0.7224 (5)	0.094 (3)	
H8A	0.3758	0.1447	0.6871	0.141*	
H8B	0.3670	0.1509	0.7801	0.141*	
H8C	0.4981	0.2026	0.7151	0.141*	
C9	−0.0869 (7)	0.7200 (4)	0.5783 (3)	0.0457 (12)	
C10	−0.1867 (9)	0.7369 (4)	0.6440 (4)	0.0701 (18)	
H10A	−0.2013	0.6857	0.6906	0.084*	
C11	−0.2681 (10)	0.8297 (5)	0.6430 (5)	0.0770 (19)	
H11A	−0.3370	0.8393	0.6889	0.092*	
C12	−0.2492 (9)	0.9063 (4)	0.5768 (5)	0.0673 (18)	
C13	−0.1481 (13)	0.8886 (5)	0.5111 (5)	0.090 (3)	
H13A	−0.1320	0.9404	0.4652	0.108*	
C14	−0.0678 (12)	0.7970 (4)	0.5095 (4)	0.083 (2)	
H14A	−0.0020	0.7874	0.4627	0.099*	
C15	−0.3410 (12)	1.0074 (5)	0.5779 (6)	0.097 (3)	
H15A	−0.3125	1.0535	0.5271	0.145*	
H15B	−0.3041	1.0230	0.6255	0.145*	
H15C	−0.4641	1.0093	0.5819	0.145*	
C16	0.2408 (7)	0.0397 (4)	1.0044 (3)	0.0476 (12)	
C17	0.1778 (6)	0.1389 (3)	0.9681 (3)	0.0394 (10)	
C18	0.1523 (7)	0.1990 (4)	1.0221 (3)	0.0465 (12)	
C19	0.1914 (9)	0.1636 (5)	1.1055 (4)	0.0627 (16)	
H19A	0.1752	0.2049	1.1397	0.075*	
C20	0.2555 (10)	0.0657 (5)	1.1392 (4)	0.0711 (18)	
H20A	0.2793	0.0420	1.1962	0.085*	
C21	0.2832 (10)	0.0052 (4)	1.0904 (4)	0.0668 (17)	
H21A	0.3302	−0.0594	1.1132	0.080*	
C22	0.2596 (8)	−0.0269 (4)	0.9567 (3)	0.0538 (14)	
H22A	0.3112	−0.0900	0.9825	0.065*	
C23	0.0593 (17)	0.3594 (6)	1.0325 (7)	0.144 (5)	
H23A	−0.0264	0.3413	1.0756	0.216*	
H23B	0.0190	0.4231	0.9974	0.216*	
H23C	0.1656	0.3585	1.0580	0.216*	
C24	0.2346 (7)	−0.0733 (3)	0.8296 (3)	0.0452 (12)	
C25	0.1500 (8)	−0.0456 (4)	0.7550 (4)	0.0589 (15)	
H25A	0.0786	0.0144	0.7385	0.071*	
C26	0.1708 (9)	−0.1065 (4)	0.7045 (4)	0.0658 (17)	
H26A	0.1108	−0.0877	0.6545	0.079*	
C27	0.2795 (8)	−0.1956 (4)	0.7264 (4)	0.0582 (15)	
C28	0.3595 (9)	−0.2217 (4)	0.8015 (4)	0.0663 (17)	
H28A	0.4299	−0.2819	0.8185	0.080*	
C29	0.3398 (8)	−0.1621 (4)	0.8528 (4)	0.0598 (15)	
H29A	0.3976	−0.1816	0.9035	0.072*	
C30	0.3049 (11)	−0.2602 (6)	0.6691 (6)	0.090 (2)	
H30A	0.3798	−0.3195	0.6951	0.136*	
H30B	0.3564	−0.2287	0.6167	0.136*	
H30C	0.1948	−0.2738	0.6589	0.136*	
C31	0.3016 (13)	0.4851 (7)	0.7970 (8)	0.128 (4)	
H31A	0.3892	0.4917	0.8313	0.192*	
H31B	0.2024	0.5346	0.7965	0.192*	
H31C	0.3464	0.4912	0.7407	0.192*	

### Atomic displacement parameters (Å^2^)

**Table:**

	*U*^11^	*U*^22^	*U*^33^	*U*^12^	*U*^13^	*U*^23^
Sm	0.04454 (18)	0.03294 (16)	0.05411 (19)	−0.00013 (11)	−0.00159 (12)	−0.00623 (12)
O1	0.047 (2)	0.0330 (17)	0.053 (2)	−0.0016 (15)	0.0119 (16)	−0.0051 (15)
O2	0.049 (2)	0.0330 (18)	0.078 (3)	0.0069 (16)	0.0148 (19)	−0.0046 (18)
O3	0.050 (2)	0.0344 (17)	0.046 (2)	0.0045 (15)	−0.0124 (16)	−0.0070 (15)
O4	0.077 (3)	0.039 (2)	0.070 (3)	0.0017 (18)	−0.020 (2)	−0.0224 (19)
O5	0.097 (15)	0.072 (13)	0.18 (2)	0.028 (9)	−0.043 (14)	−0.074 (14)
O5'	0.090 (12)	0.049 (8)	0.084 (9)	0.020 (6)	−0.034 (8)	−0.036 (7)
O6	0.108 (4)	0.063 (3)	0.083 (4)	0.012 (3)	0.012 (3)	−0.015 (3)
O7	0.116 (5)	0.069 (3)	0.133 (5)	0.048 (3)	−0.016 (4)	−0.049 (3)
O8	0.056 (3)	0.086 (3)	0.071 (3)	−0.006 (2)	−0.004 (2)	−0.001 (3)
O9	0.059 (3)	0.080 (3)	0.068 (3)	−0.016 (2)	−0.002 (2)	0.004 (2)
O10	0.041 (2)	0.099 (4)	0.090 (3)	−0.014 (2)	0.003 (2)	0.017 (3)
O11	0.072 (3)	0.0380 (19)	0.062 (2)	0.0004 (18)	−0.021 (2)	−0.0105 (18)
O12	0.075 (3)	0.047 (2)	0.044 (2)	−0.0038 (19)	−0.0061 (18)	−0.0042 (17)
O13	0.076 (3)	0.084 (3)	0.072 (3)	−0.017 (2)	−0.008 (2)	−0.039 (3)
O14	0.060 (3)	0.064 (3)	0.113 (4)	−0.023 (2)	−0.036 (3)	0.014 (3)
N1	0.049 (2)	0.032 (2)	0.043 (2)	−0.0102 (18)	−0.0021 (18)	−0.0069 (17)
N2	0.055 (3)	0.029 (2)	0.047 (2)	0.0057 (18)	−0.001 (2)	−0.0069 (18)
N3	0.068 (4)	0.043 (3)	0.093 (4)	0.012 (3)	−0.006 (3)	−0.023 (3)
N4	0.042 (3)	0.043 (2)	0.050 (3)	−0.0067 (19)	−0.002 (2)	0.007 (2)
N5	0.042 (2)	0.054 (3)	0.049 (3)	−0.004 (2)	0.0041 (19)	−0.019 (2)
C1	0.037 (3)	0.045 (3)	0.045 (3)	−0.014 (2)	−0.003 (2)	−0.015 (2)
C2	0.036 (3)	0.040 (3)	0.051 (3)	−0.011 (2)	0.003 (2)	−0.017 (2)
C3	0.038 (3)	0.038 (3)	0.064 (3)	−0.007 (2)	0.006 (2)	−0.016 (2)
C4	0.047 (3)	0.051 (3)	0.073 (4)	−0.004 (2)	0.006 (3)	−0.031 (3)
C5	0.055 (3)	0.068 (4)	0.058 (3)	−0.015 (3)	0.014 (3)	−0.035 (3)
C6	0.053 (3)	0.062 (3)	0.042 (3)	−0.019 (3)	0.005 (2)	−0.019 (3)
C7	0.049 (3)	0.042 (3)	0.040 (3)	−0.015 (2)	−0.007 (2)	−0.006 (2)
C8	0.077 (5)	0.060 (4)	0.106 (6)	0.029 (4)	0.025 (4)	0.005 (4)
C9	0.048 (3)	0.039 (3)	0.053 (3)	−0.011 (2)	−0.008 (2)	−0.014 (2)
C10	0.086 (5)	0.043 (3)	0.076 (4)	−0.015 (3)	0.022 (4)	−0.011 (3)
C11	0.080 (5)	0.059 (4)	0.095 (5)	−0.005 (3)	0.015 (4)	−0.035 (4)
C12	0.079 (4)	0.039 (3)	0.088 (5)	−0.005 (3)	−0.024 (4)	−0.022 (3)
C13	0.152 (8)	0.041 (3)	0.065 (4)	−0.001 (4)	0.005 (5)	−0.005 (3)
C14	0.137 (7)	0.046 (3)	0.051 (4)	0.002 (4)	0.013 (4)	−0.007 (3)
C15	0.123 (7)	0.051 (4)	0.121 (7)	0.005 (4)	−0.024 (6)	−0.040 (4)
C16	0.054 (3)	0.037 (3)	0.043 (3)	−0.002 (2)	0.000 (2)	−0.003 (2)
C17	0.035 (3)	0.037 (2)	0.043 (3)	−0.002 (2)	−0.002 (2)	−0.009 (2)
C18	0.043 (3)	0.040 (3)	0.056 (3)	−0.005 (2)	−0.005 (2)	−0.013 (2)
C19	0.077 (4)	0.063 (4)	0.052 (3)	−0.010 (3)	−0.001 (3)	−0.023 (3)
C20	0.104 (5)	0.061 (4)	0.040 (3)	−0.009 (4)	−0.006 (3)	−0.005 (3)
C21	0.098 (5)	0.046 (3)	0.043 (3)	−0.001 (3)	−0.004 (3)	0.001 (3)
C22	0.069 (4)	0.032 (3)	0.051 (3)	0.004 (2)	0.004 (3)	−0.006 (2)
C23	0.230 (13)	0.064 (5)	0.150 (9)	0.048 (6)	−0.099 (9)	−0.072 (6)
C24	0.047 (3)	0.032 (2)	0.054 (3)	0.002 (2)	0.000 (2)	−0.013 (2)
C25	0.072 (4)	0.036 (3)	0.063 (4)	0.011 (3)	−0.011 (3)	−0.015 (3)
C26	0.078 (4)	0.054 (3)	0.065 (4)	−0.002 (3)	−0.019 (3)	−0.016 (3)
C27	0.056 (3)	0.049 (3)	0.074 (4)	−0.001 (3)	−0.001 (3)	−0.029 (3)
C28	0.072 (4)	0.041 (3)	0.081 (4)	0.018 (3)	−0.013 (3)	−0.024 (3)
C29	0.063 (4)	0.048 (3)	0.061 (3)	0.013 (3)	−0.010 (3)	−0.014 (3)
C30	0.092 (6)	0.074 (5)	0.122 (7)	0.004 (4)	−0.012 (5)	−0.062 (5)
C31	0.106 (7)	0.085 (6)	0.190 (11)	−0.044 (5)	−0.050 (7)	−0.006 (7)

### Geometric parameters (Å, °)

**Table:**

Sm—O3	2.428 (3)	C8—H8B	0.9600
Sm—O1	2.486 (3)	C8—H8C	0.9600
Sm—O14	2.529 (4)	C9—C10	1.349 (8)
Sm—O5	2.59 (2)	C9—C14	1.380 (8)
Sm—O5'	2.594 (19)	C10—C11	1.389 (9)
Sm—O11	2.648 (4)	C10—H10A	0.9300
Sm—O8	2.660 (4)	C11—C12	1.352 (10)
Sm—O12	2.676 (4)	C11—H11A	0.9300
Sm—O9	2.688 (4)	C12—C13	1.357 (11)
Sm—O6	2.743 (5)	C12—C15	1.530 (9)
Sm—O2	2.806 (4)	C13—C14	1.382 (9)
Sm—O4	2.957 (4)	C13—H13A	0.9300
O1—C2	1.283 (6)	C14—H14A	0.9300
O2—C3	1.387 (7)	C15—H15A	0.9600
O2—C8	1.423 (7)	C15—H15B	0.9600
O3—C17	1.288 (6)	C15—H15C	0.9600
O4—C18	1.359 (6)	C16—C22	1.407 (7)
O4—C23	1.434 (8)	C16—C21	1.413 (8)
O5—N3	1.12 (2)	C16—C17	1.416 (7)
O5'—N3	1.354 (19)	C17—C18	1.407 (7)
O6—N3	1.179 (7)	C18—C19	1.368 (8)
O7—N3	1.229 (7)	C19—C20	1.396 (9)
O8—N4	1.234 (6)	C19—H19A	0.9300
O9—N4	1.208 (6)	C20—C21	1.344 (9)
O10—N4	1.227 (6)	C20—H20A	0.9300
O11—N5	1.238 (6)	C21—H21A	0.9300
O12—N5	1.246 (6)	C22—H22A	0.9300
O13—N5	1.227 (6)	C23—H23A	0.9600
O14—C31	1.431 (9)	C23—H23B	0.9600
O14—H14B	0.8200	C23—H23C	0.9600
N1—C7	1.307 (7)	C24—C25	1.368 (8)
N1—C9	1.417 (6)	C24—C29	1.375 (7)
N1—H1A	0.8601	C25—C26	1.371 (8)
N2—C22	1.285 (7)	C25—H25A	0.9300
N2—C24	1.427 (6)	C26—C27	1.390 (8)
N2—H2A	0.8601	C26—H26A	0.9300
C1—C6	1.407 (7)	C27—C28	1.360 (9)
C1—C7	1.415 (7)	C27—C30	1.503 (8)
C1—C2	1.421 (7)	C28—C29	1.367 (8)
C2—C3	1.431 (7)	C28—H28A	0.9300
C3—C4	1.359 (8)	C29—H29A	0.9300
C4—C5	1.389 (9)	C30—H30A	0.9600
C4—H4A	0.9300	C30—H30B	0.9600
C5—C6	1.354 (8)	C30—H30C	0.9600
C5—H5A	0.9300	C31—H31A	0.9600
C6—H6A	0.9300	C31—H31B	0.9600
C7—H7A	0.9300	C31—H31C	0.9600
C8—H8A	0.9600		
			
O3—Sm—O1	131.61 (12)	O1—C2—C1	122.9 (4)
O3—Sm—O14	83.87 (13)	O1—C2—C3	120.6 (5)
O1—Sm—O14	70.74 (14)	C1—C2—C3	116.5 (5)
O3—Sm—O5	126.1 (6)	C4—C3—O2	126.3 (5)
O1—Sm—O5	83.5 (5)	C4—C3—C2	120.8 (5)
O14—Sm—O5	70.3 (5)	O2—C3—C2	112.8 (4)
O3—Sm—O5'	117.5 (4)	C3—C4—C5	121.4 (5)
O1—Sm—O5'	99.2 (3)	C3—C4—H4A	119.3
O14—Sm—O5'	81.8 (4)	C5—C4—H4A	119.3
O5—Sm—O5'	17.0 (6)	C6—C5—C4	120.2 (5)
O3—Sm—O11	64.60 (11)	C6—C5—H5A	119.9
O1—Sm—O11	108.63 (12)	C4—C5—H5A	119.9
O14—Sm—O11	137.26 (15)	C5—C6—C1	120.3 (5)
O5—Sm—O11	151.9 (5)	C5—C6—H6A	119.8
O5'—Sm—O11	137.5 (4)	C1—C6—H6A	119.8
O3—Sm—O8	109.06 (14)	N1—C7—C1	124.8 (5)
O1—Sm—O8	112.92 (13)	N1—C7—H7A	117.6
O14—Sm—O8	151.18 (19)	C1—C7—H7A	117.6
O5—Sm—O8	81.5 (5)	O2—C8—H8A	109.5
O5'—Sm—O8	69.4 (4)	O2—C8—H8B	109.5
O11—Sm—O8	70.40 (15)	H8A—C8—H8B	109.5
O3—Sm—O12	109.27 (11)	O2—C8—H8C	109.5
O1—Sm—O12	68.65 (12)	H8A—C8—H8C	109.5
O14—Sm—O12	134.84 (16)	H8B—C8—H8C	109.5
O5—Sm—O12	122.6 (6)	C10—C9—C14	118.6 (5)
O5'—Sm—O12	123.4 (4)	C10—C9—N1	119.1 (5)
O11—Sm—O12	46.92 (11)	C14—C9—N1	122.3 (5)
O8—Sm—O12	66.47 (14)	C9—C10—C11	121.0 (6)
O3—Sm—O9	69.41 (13)	C9—C10—H10A	119.5
O1—Sm—O9	158.08 (13)	C11—C10—H10A	119.5
O14—Sm—O9	124.08 (16)	C12—C11—C10	121.4 (7)
O5—Sm—O9	87.0 (5)	C12—C11—H11A	119.3
O5'—Sm—O9	70.0 (4)	C10—C11—H11A	119.3
O11—Sm—O9	72.44 (15)	C11—C12—C13	117.2 (6)
O8—Sm—O9	45.88 (14)	C11—C12—C15	119.9 (7)
O12—Sm—O9	100.68 (14)	C13—C12—C15	122.9 (7)
O3—Sm—O6	164.80 (15)	C12—C13—C14	122.9 (7)
O1—Sm—O6	62.02 (14)	C12—C13—H13A	118.6
O14—Sm—O6	96.90 (17)	C14—C13—H13A	118.6
O5—Sm—O6	41.8 (6)	C9—C14—C13	119.0 (7)
O5'—Sm—O6	48.0 (4)	C9—C14—H14A	120.5
O11—Sm—O6	121.16 (16)	C13—C14—H14A	120.5
O8—Sm—O6	63.90 (17)	C12—C15—H15A	109.5
O12—Sm—O6	80.97 (16)	C12—C15—H15B	109.5
O9—Sm—O6	98.12 (16)	H15A—C15—H15B	109.5
O3—Sm—O2	74.45 (12)	C12—C15—H15C	109.5
O1—Sm—O2	59.20 (10)	H15A—C15—H15C	109.5
O14—Sm—O2	73.05 (17)	H15B—C15—H15C	109.5
O5—Sm—O2	134.4 (5)	C22—C16—C21	118.4 (5)
O5'—Sm—O2	151.0 (4)	C22—C16—C17	121.2 (5)
O11—Sm—O2	71.23 (13)	C21—C16—C17	120.4 (5)
O8—Sm—O2	134.55 (14)	O3—C17—C18	120.4 (4)
O12—Sm—O2	69.76 (13)	O3—C17—C16	122.5 (4)
O9—Sm—O2	136.84 (13)	C18—C17—C16	117.1 (4)
O6—Sm—O2	120.37 (14)	O4—C18—C19	125.2 (5)
O3—Sm—O4	56.71 (10)	O4—C18—C17	113.3 (4)
O1—Sm—O4	131.51 (12)	C19—C18—C17	121.5 (5)
O14—Sm—O4	62.62 (15)	C18—C19—C20	120.1 (5)
O5—Sm—O4	69.4 (6)	C18—C19—H19A	119.9
O5'—Sm—O4	62.8 (4)	C20—C19—H19A	119.9
O11—Sm—O4	114.13 (11)	C21—C20—C19	120.9 (6)
O8—Sm—O4	102.27 (13)	C21—C20—H20A	119.6
O12—Sm—O4	159.57 (12)	C19—C20—H20A	119.6
O9—Sm—O4	61.61 (13)	C20—C21—C16	120.0 (5)
O6—Sm—O4	110.19 (15)	C20—C21—H21A	120.0
O2—Sm—O4	115.10 (12)	C16—C21—H21A	120.0
C2—O1—Sm	129.3 (3)	N2—C22—C16	125.5 (5)
C3—O2—C8	116.8 (5)	N2—C22—H22A	117.2
C3—O2—Sm	117.3 (3)	C16—C22—H22A	117.2
C8—O2—Sm	125.4 (4)	O4—C23—H23A	109.5
C17—O3—Sm	133.4 (3)	O4—C23—H23B	109.5
C18—O4—C23	117.3 (5)	H23A—C23—H23B	109.5
C18—O4—Sm	114.4 (3)	O4—C23—H23C	109.5
C23—O4—Sm	127.9 (4)	H23A—C23—H23C	109.5
N3—O5—Sm	104.8 (14)	H23B—C23—H23C	109.5
N3—O5'—Sm	97.3 (9)	C25—C24—C29	119.3 (5)
N3—O6—Sm	94.8 (4)	C25—C24—N2	118.5 (4)
N4—O8—Sm	98.8 (3)	C29—C24—N2	122.1 (5)
N4—O9—Sm	98.1 (3)	C24—C25—C26	119.8 (5)
N5—O11—Sm	98.8 (3)	C24—C25—H25A	120.1
N5—O12—Sm	97.1 (3)	C26—C25—H25A	120.1
C31—O14—Sm	131.7 (5)	C25—C26—C27	121.5 (6)
C31—O14—H14B	109.5	C25—C26—H26A	119.2
Sm—O14—H14B	118.5	C27—C26—H26A	119.2
C7—N1—C9	127.4 (4)	C28—C27—C26	117.3 (5)
C7—N1—H1A	116.2	C28—C27—C30	121.9 (6)
C9—N1—H1A	116.4	C26—C27—C30	120.8 (6)
C22—N2—C24	126.7 (4)	C27—C28—C29	122.0 (5)
C22—N2—H2A	116.5	C27—C28—H28A	119.0
C24—N2—H2A	116.8	C29—C28—H28A	119.0
O5—N3—O6	111.8 (13)	C28—C29—C24	120.1 (5)
O5—N3—O7	123.0 (13)	C28—C29—H29A	120.0
O6—N3—O7	122.2 (7)	C24—C29—H29A	120.0
O5—N3—O5'	34.4 (13)	C27—C30—H30A	109.5
O6—N3—O5'	118.1 (9)	C27—C30—H30B	109.5
O7—N3—O5'	117.3 (10)	H30A—C30—H30B	109.5
O9—N4—O10	121.5 (5)	C27—C30—H30C	109.5
O9—N4—O8	117.2 (5)	H30A—C30—H30C	109.5
O10—N4—O8	121.3 (5)	H30B—C30—H30C	109.5
O13—N5—O11	120.7 (5)	O14—C31—H31A	109.5
O13—N5—O12	122.1 (5)	O14—C31—H31B	109.5
O11—N5—O12	117.2 (4)	H31A—C31—H31B	109.5
C6—C1—C7	119.2 (5)	O14—C31—H31C	109.5
C6—C1—C2	120.7 (5)	H31A—C31—H31C	109.5
C7—C1—C2	120.1 (5)	H31B—C31—H31C	109.5
			
O3—Sm—O1—C2	26.1 (5)	O3—Sm—O11—N5	−161.7 (4)
O14—Sm—O1—C2	88.6 (4)	O1—Sm—O11—N5	−33.7 (3)
O5—Sm—O1—C2	160.0 (7)	O14—Sm—O11—N5	−115.1 (3)
O5'—Sm—O1—C2	166.5 (6)	O5—Sm—O11—N5	78.5 (13)
O11—Sm—O1—C2	−46.1 (4)	O5'—Sm—O11—N5	94.5 (6)
O8—Sm—O1—C2	−122.1 (4)	O8—Sm—O11—N5	74.8 (3)
O12—Sm—O1—C2	−71.2 (4)	O12—Sm—O11—N5	−0.9 (3)
O9—Sm—O1—C2	−135.2 (4)	O9—Sm—O11—N5	123.3 (3)
O6—Sm—O1—C2	−162.1 (5)	O6—Sm—O11—N5	34.3 (4)
O2—Sm—O1—C2	7.3 (4)	O2—Sm—O11—N5	−80.4 (3)
O4—Sm—O1—C2	104.9 (4)	O4—Sm—O11—N5	169.8 (3)
O3—Sm—O2—C3	−173.2 (4)	O3—Sm—O12—N5	19.3 (3)
O1—Sm—O2—C3	−7.7 (3)	O1—Sm—O12—N5	147.5 (3)
O14—Sm—O2—C3	−85.0 (4)	O14—Sm—O12—N5	120.1 (3)
O5—Sm—O2—C3	−47.4 (9)	O5—Sm—O12—N5	−145.7 (6)
O5'—Sm—O2—C3	−54.0 (9)	O5'—Sm—O12—N5	−125.5 (5)
O11—Sm—O2—C3	118.9 (4)	O11—Sm—O12—N5	0.9 (3)
O8—Sm—O2—C3	85.2 (4)	O8—Sm—O12—N5	−83.7 (3)
O12—Sm—O2—C3	68.9 (3)	O9—Sm—O12—N5	−52.5 (3)
O9—Sm—O2—C3	152.9 (3)	O6—Sm—O12—N5	−149.1 (3)
O6—Sm—O2—C3	3.1 (4)	O2—Sm—O12—N5	83.7 (3)
O4—Sm—O2—C3	−132.6 (3)	O4—Sm—O12—N5	−24.1 (5)
O3—Sm—O2—C8	−1.5 (5)	O3—Sm—O14—C31	172.3 (9)
O1—Sm—O2—C8	164.1 (6)	O1—Sm—O14—C31	34.1 (9)
O14—Sm—O2—C8	86.8 (6)	O5—Sm—O14—C31	−55.8 (10)
O5—Sm—O2—C8	124.3 (10)	O5'—Sm—O14—C31	−68.7 (9)
O5'—Sm—O2—C8	117.7 (10)	O11—Sm—O14—C31	131.0 (8)
O11—Sm—O2—C8	−69.4 (6)	O8—Sm—O14—C31	−68.6 (10)
O8—Sm—O2—C8	−103.1 (6)	O12—Sm—O14—C31	61.1 (9)
O12—Sm—O2—C8	−119.3 (6)	O9—Sm—O14—C31	−127.7 (9)
O9—Sm—O2—C8	−35.3 (6)	O6—Sm—O14—C31	−23.0 (9)
O6—Sm—O2—C8	174.9 (6)	O2—Sm—O14—C31	96.7 (9)
O4—Sm—O2—C8	39.1 (6)	O4—Sm—O14—C31	−132.2 (9)
O1—Sm—O3—C17	131.5 (4)	Sm—O5—N3—O6	−26.8 (14)
O14—Sm—O3—C17	74.1 (4)	Sm—O5—N3—O7	172.7 (6)
O5—Sm—O3—C17	14.0 (8)	Sm—O5—N3—O5'	81 (3)
O5'—Sm—O3—C17	−3.3 (6)	Sm—O6—N3—O5	24.4 (12)
O11—Sm—O3—C17	−135.6 (5)	Sm—O6—N3—O7	−174.9 (7)
O8—Sm—O3—C17	−79.4 (4)	Sm—O6—N3—O5'	−13.0 (11)
O12—Sm—O3—C17	−150.3 (4)	Sm—O5'—N3—O5	−74 (3)
O9—Sm—O3—C17	−55.8 (4)	Sm—O5'—N3—O6	13.9 (11)
O6—Sm—O3—C17	−19.7 (8)	Sm—O5'—N3—O7	176.6 (6)
O2—Sm—O3—C17	148.2 (4)	Sm—O9—N4—O10	−179.1 (5)
O4—Sm—O3—C17	13.0 (4)	Sm—O9—N4—O8	2.1 (6)
O3—Sm—O4—C18	−9.3 (3)	Sm—O8—N4—O9	−2.1 (6)
O1—Sm—O4—C18	−128.0 (3)	Sm—O8—N4—O10	179.1 (5)
O14—Sm—O4—C18	−110.6 (4)	Sm—O11—N5—O13	−178.6 (4)
O5—Sm—O4—C18	171.6 (6)	Sm—O11—N5—O12	1.6 (5)
O5'—Sm—O4—C18	154.5 (6)	Sm—O12—N5—O13	178.6 (4)
O11—Sm—O4—C18	21.8 (4)	Sm—O12—N5—O11	−1.6 (5)
O8—Sm—O4—C18	95.6 (4)	Sm—O1—C2—C1	173.7 (3)
O12—Sm—O4—C18	41.5 (6)	Sm—O1—C2—C3	−6.3 (7)
O9—Sm—O4—C18	73.6 (4)	C6—C1—C2—O1	−177.3 (5)
O6—Sm—O4—C18	162.0 (4)	C7—C1—C2—O1	5.3 (7)
O2—Sm—O4—C18	−57.9 (4)	C6—C1—C2—C3	2.7 (7)
O3—Sm—O4—C23	178.3 (8)	C7—C1—C2—C3	−174.8 (4)
O1—Sm—O4—C23	59.7 (8)	C8—O2—C3—C4	15.5 (9)
O14—Sm—O4—C23	77.0 (8)	Sm—O2—C3—C4	−172.0 (4)
O5—Sm—O4—C23	−0.8 (9)	C8—O2—C3—C2	−164.4 (6)
O5'—Sm—O4—C23	−17.9 (9)	Sm—O2—C3—C2	8.1 (5)
O11—Sm—O4—C23	−150.6 (8)	O1—C2—C3—C4	177.7 (5)
O8—Sm—O4—C23	−76.8 (8)	C1—C2—C3—C4	−2.2 (7)
O12—Sm—O4—C23	−130.8 (8)	O1—C2—C3—O2	−2.4 (7)
O9—Sm—O4—C23	−98.7 (8)	C1—C2—C3—O2	177.7 (4)
O6—Sm—O4—C23	−10.3 (8)	O2—C3—C4—C5	−179.4 (5)
O2—Sm—O4—C23	129.8 (8)	C2—C3—C4—C5	0.6 (8)
O3—Sm—O5—N3	−151.7 (11)	C3—C4—C5—C6	0.7 (9)
O1—Sm—O5—N3	70.1 (14)	C4—C5—C6—C1	−0.3 (8)
O14—Sm—O5—N3	142.0 (15)	C7—C1—C6—C5	176.0 (5)
O5'—Sm—O5—N3	−87 (3)	C2—C1—C6—C5	−1.5 (7)
O11—Sm—O5—N3	−48 (2)	C9—N1—C7—C1	176.3 (4)
O8—Sm—O5—N3	−44.3 (13)	C6—C1—C7—N1	179.1 (5)
O12—Sm—O5—N3	10.7 (17)	C2—C1—C7—N1	−3.5 (7)
O9—Sm—O5—N3	−90.1 (14)	C7—N1—C9—C10	−175.6 (5)
O6—Sm—O5—N3	15.7 (9)	C7—N1—C9—C14	3.0 (8)
O2—Sm—O5—N3	103.7 (13)	C14—C9—C10—C11	−0.4 (10)
O4—Sm—O5—N3	−150.8 (15)	N1—C9—C10—C11	178.3 (6)
O3—Sm—O5'—N3	178.7 (7)	C9—C10—C11—C12	−0.5 (11)
O1—Sm—O5'—N3	31.2 (9)	C10—C11—C12—C13	0.3 (11)
O14—Sm—O5'—N3	100.1 (9)	C10—C11—C12—C15	179.9 (7)
O5—Sm—O5'—N3	54 (3)	C11—C12—C13—C14	0.9 (13)
O11—Sm—O5'—N3	−99.8 (8)	C15—C12—C13—C14	−178.8 (8)
O8—Sm—O5'—N3	−79.9 (9)	C10—C9—C14—C13	1.5 (11)
O12—Sm—O5'—N3	−39.2 (10)	N1—C9—C14—C13	−177.1 (7)
O9—Sm—O5'—N3	−129.0 (10)	C12—C13—C14—C9	−1.8 (13)
O6—Sm—O5'—N3	−7.0 (6)	Sm—O3—C17—C18	−15.1 (7)
O2—Sm—O5'—N3	70.2 (14)	Sm—O3—C17—C16	165.9 (4)
O4—Sm—O5'—N3	163.4 (10)	C22—C16—C17—O3	−5.2 (8)
O3—Sm—O6—N3	27.7 (9)	C21—C16—C17—O3	176.1 (5)
O1—Sm—O6—N3	−128.2 (5)	C22—C16—C17—C18	175.8 (5)
O14—Sm—O6—N3	−64.3 (5)	C21—C16—C17—C18	−2.9 (8)
O5—Sm—O6—N3	−14.5 (8)	C23—O4—C18—C19	−0.3 (10)
O5'—Sm—O6—N3	8.0 (6)	Sm—O4—C18—C19	−173.5 (5)
O11—Sm—O6—N3	136.0 (4)	C23—O4—C18—C17	−179.7 (7)
O8—Sm—O6—N3	93.1 (5)	Sm—O4—C18—C17	7.1 (5)
O12—Sm—O6—N3	161.3 (5)	O3—C17—C18—O4	2.1 (7)
O9—Sm—O6—N3	61.7 (5)	C16—C17—C18—O4	−178.8 (5)
O2—Sm—O6—N3	−138.7 (4)	O3—C17—C18—C19	−177.3 (5)
O4—Sm—O6—N3	−1.0 (5)	C16—C17—C18—C19	1.8 (8)
O3—Sm—O8—N4	32.6 (4)	O4—C18—C19—C20	179.7 (6)
O1—Sm—O8—N4	−172.0 (3)	C17—C18—C19—C20	−1.0 (10)
O14—Sm—O8—N4	−80.6 (5)	C18—C19—C20—C21	1.4 (11)
O5—Sm—O8—N4	−92.8 (7)	C19—C20—C21—C16	−2.5 (11)
O5'—Sm—O8—N4	−80.5 (5)	C22—C16—C21—C20	−175.4 (7)
O11—Sm—O8—N4	85.4 (4)	C17—C16—C21—C20	3.3 (10)
O12—Sm—O8—N4	135.9 (4)	C24—N2—C22—C16	177.6 (5)
O9—Sm—O8—N4	1.2 (3)	C21—C16—C22—N2	173.2 (6)
O6—Sm—O8—N4	−132.8 (4)	C17—C16—C22—N2	−5.5 (9)
O2—Sm—O8—N4	119.3 (3)	C22—N2—C24—C25	169.1 (6)
O4—Sm—O8—N4	−26.1 (4)	C22—N2—C24—C29	−11.6 (9)
O3—Sm—O9—N4	−149.4 (4)	C29—C24—C25—C26	0.1 (10)
O1—Sm—O9—N4	15.7 (6)	N2—C24—C25—C26	179.4 (6)
O14—Sm—O9—N4	143.6 (3)	C24—C25—C26—C27	−1.4 (11)
O5—Sm—O9—N4	80.0 (7)	C25—C26—C27—C28	2.4 (10)
O5'—Sm—O9—N4	79.1 (6)	C25—C26—C27—C30	−178.2 (7)
O11—Sm—O9—N4	−80.7 (4)	C26—C27—C28—C29	−2.1 (11)
O8—Sm—O9—N4	−1.2 (3)	C30—C27—C28—C29	178.5 (7)
O12—Sm—O9—N4	−42.7 (4)	C27—C28—C29—C24	0.9 (11)
O6—Sm—O9—N4	39.6 (4)	C25—C24—C29—C28	0.2 (10)
O2—Sm—O9—N4	−114.4 (4)	N2—C24—C29—C28	−179.1 (6)
O4—Sm—O9—N4	148.2 (4)		

### Hydrogen-bond geometry (Å, °)

**Table:**

*D*—H···*A*	*D*—H	H···*A*	*D*···*A*	*D*—H···*A*
O14—H14B···O10^i^	0.82	2.04	2.859 (6)	174
O14—H14B···O8^i^	0.82	2.53	3.121 (6)	130
O14—H14B···N4^i^	0.82	2.60	3.367 (6)	157
N1—H1A···O1	0.86	1.96	2.637 (5)	135
N1—H1A···O6	0.86	2.65	3.449 (7)	154
N2—H2A···O3	0.86	2.02	2.678 (5)	132
N2—H2A···O11	0.86	2.52	3.311 (5)	153

Symmetry codes: (i) *x*+1, *y*, *z*.

## References

[bb1] Bruker (2006). *APEX2* and *SAINT* Bruker AXS Inc., Madison, Wisconsin, USA.

[bb2] Burrows, R. C. & Bailar, J. C. (1966). *J. Am. Chem. Soc.* **88**, 4150–4152.

[bb3] Leadbeater, N. E. & Marco, M. (2002). *Chem. Rev.* **102**, 3217–3273. 10.1021/cr010361c 12371884

[bb4] Li, H.-Q., Xian, H.-D., Liu, J.-F. & Zhao, G.-L. (2008). *Acta Cryst.* E**64**, m1593–m1594. 10.1107/S1600536808038099 PMC2959993 21581192

[bb5] Liu, J.-L., Cai, H.-T. & Zhao, G.-L. (2010). *Acta Cryst.* E**66**, m1332–m1333. 10.1107/S1600536810038109 PMC2983206 21587463

[bb6] Liu, J.-F., Liu, J.-L. & Zhao, G.-L. (2009). *Acta Cryst.* E**65**, m1385–m1386. 10.1107/S1600536809041361 PMC2971141 21578132

[bb7] Quici, S., Marzanni, G., Forni, A., Accorsi, G. & Barigelletti, F. (2004). *Inorg. Chem.* **43**, 1294–1301. 10.1021/ic035143b 14966964

[bb8] Sheldrick, G. M. (1996). *SADABS* University of Göttingen, Germany.

[bb9] Sheldrick, G. M. (2008). *Acta Cryst.* A**64**, 112–122. 10.1107/S0108767307043930 18156677

[bb10] Xian, H.-D., Liu, J.-F., Li, H.-Q. & Zhao, G.-L. (2008). *Acta Cryst.* E**64**, m1422. 10.1107/S1600536808033102 PMC2959557 21580867

[bb11] Zhao, G.-L., Zhang, P.-H. & Feng, Y.-L. (2005). *Chin. J. Inorg. Chem.* **21**, 421–424.

